# NLRP3 inflammasome inhibitor MCC950 attenuates primary dysmenorrhea in mice via the NF-κB/COX-2/PG pathway

**DOI:** 10.1186/s12950-020-00251-7

**Published:** 2020-06-24

**Authors:** Biao Tang, Dan Liu, Lingyu Chen, Yu Liu

**Affiliations:** 1 Department of Physiology, Medical School, Hunan University of Chinese Medicine, No.300 Xueshi Road, Changsa, China; 2 Hanpu Science & Education District, Hunan province, Changsha, 410208 China

**Keywords:** Primary dysmenorrhea, Nucleotide-binding oligomerization domain-like receptor protein 3, Nuclear factor kappa B, Cyclooxygenase-2, Prostaglandins

## Abstract

**Background:**

Primary dysmenorrhea (PD) constitutes a common gynecological disease among young women. The NLRP3 inflammasome may be activated and expressed in PD, but the mechanistic link between NLRP3 inflammasome activation and PD is still unclear.

**Methods:**

To investigate the potential role of NLRP3 inflammasome activation in the pathogenesis of PD, 30 female Kunming mice without pregnancy were used for experiments. The PD mouse model was constructed by 11 days of successive co-treatment with estradiol and oxytocin. MCC950, a potent and specific small-molecule inhibitor of the NLRP3 inflammasome, was used to treat PD mice. The disease level was assessed by the writhing response and hot water tail-flick test. The levels of prostaglandin E_2_ (PGE_2_) and prostaglandin F_2_ alpha (PGF_2α_) in the uterine tissues of mice were detected by ELISA. The expression levels of protein and cytokines, including NLRP3, cysteine aspartic acid-specific protease 1 (caspase-1), interleukin (IL)-1β, IL-18, nuclear factor kappa B (NF-κB) p65, phospho-NF-κB p65, and cyclooxygenase-2 (COX-2) were revealed by western blot analysis.

**Results:**

MCC950 greatly ameliorated the writhing response induced by the combination of oxytocin and estradiol, with an increasing length of tail-flick latency. MCC950 also significantly decreased the levels of PGF_2α_ and PGE_2_, and the expressions of NLRP3, caspase-1, IL-1β, IL-18, phospho-NF-κB p65, NF-κB p65, and COX-2 in the uterus.

**Conclusions:**

MCC950 markedly alleviated the pain and pathological damage in PD mice by inhibiting NLRP3 activation. The underlying mechanism may be related to hypoactive uterine inflammation via suppression of NLRP3 activation and the NF-κB/COX-2/PG pathway in uteruses of PD mice.

## Background

Primary dysmenorrhea (PD), a common gynecological disease among young women, constitutes functional dysmenorrhea without a definite gynecological pathological origin and seriously affects every aspect of the lives, work, and study of female patients [1]. Clinically, the main symptom of PD is menstrual pain.

Currently, excessive secretion of prostaglandins (PG) in uterine tissues has been proposed as a trigger for PD, though the mechanisms responsible for PD remain poorly understood [2]. Cyclooxygenase-2 (COX-2), a key enzyme involved in PG production, has its expression dramatically enhanced in the period of pre-menstruation. Subsequently, increased COX-2 converts arachidonic acid to PG, which induces spastic pain [3]. Nuclear factor kappa B (NF-κB) is a crucial factor for the regulation of COX-2 [4]. Increasing studies have shown that the NF-κB/COX-2/PG pathway plays a pathogenic role in PD, which suggests that therapeutic interventions of the NF-κB/COX-2/PG pathway can exert protective effects against PD [5, 6].

It has been reported that inflammation potently mediates the pathological process of PD. Activated macrophages produce a wide array of inflammatory cytokines, such as interleukin-1β (IL-1β), IL-6, and tumor necrosis factor α, which stimulate the synthesis and release of PG, leading to excessive contraction of uterine smooth muscles and ischemic injury in PD [7, 8]. As a core part of inflammation, the nucleotide-binding oligomerization domain-like receptor protein 3 (NLRP3) inflammasome is involved in a wide array of acute and chronic diseases, such as coronary heart disease, stroke, pneumonia, acute kidney injury, and viral hepatitis [9]. The assembly of the NLRP3 inflammasome as stimulated by NLRP3 irritants triggers proteolytic cleavage of dormant procaspase-1 into active caspase-1, which converts the cytokines pro-IL-1β and pro-IL-18 into mature and biologically active IL-1β and IL-18, respectively, leading to a cascade of deleterious inflammation [10]. Moreover, there is increasing evidence indicating that the NLRP3 inflammasome plays a regulatory role in the expression of COX-2 via the NF-κB pathway [11, 12]. Our previous studies have found that the NLRP3 inflammasome can be activated and expressed in PD, and effective traditional electroacupuncture therapy to treat PD is associated with the inhibition of NLRP3 inflammasome activation [13]. However, the mechanistic link between NLRP3 inflammasome activation and PD is still unclear. Therefore, we constructed a PD mouse model to investigate whether inhibition of NLRP3 inflammasomes through the NF-κB/COX-2/PG pathway exerts protective effects against PD induced by a combination of estradiol and oxytocin.

## Materials and methods

### Animals

Thirty healthy female nonpregnant Kunming mice (18–22 g, 6–8 weeks old) in the same cycle were purchased from Hunan Slack Jingda Lab Animal Co., Ltd. [Hunan, China, SYXK (Xiang) 2013–0004] All animals were kept on natural circadian rhythms at optimal temperature (20 °C–25 °C) and humidity (50–70%), with free access to water and food. After a week, the 30 mice were divided into three groups (Saline group, PD group, and MCC950 group), with 10 mice in each group. All experimental procedures were approved by the Instructive Notions with Respect to Caring for Laboratory Animals promulgated by the Ministry of Science and Technology of China in 2006.

### Pharmaceutical and main reagents

Oxytocin was purchased from Shanghai Hefeng Pharmaceutical Co., Ltd. (No. H31020850, Shanghai, China), and estradiol (No. J20130009, Bayer, Germany) was purchased from Bayer. Both are approved by the State Food and Drug Administration (SFDA). Other reagents and materials included MCC950 (CP-456773, Selleck, USA; CAS number: 210826–40-7; formula: C20H24N2O5S; molecular weight: 404.48 g/mol), mouse PGF_2α_ kit (production lot number: 516011, Cayman Chemical, USA), mouse PGE_2_ kit (production batch number: 514010, Cayman Chemical, USA), BCA protein quantification kit (production lot number: A53225, Thermo, USA), NLRP3 antibody (production batch number: NBP2–12446, Novus Biological, USA), phospho-NF-κB-p65 antibody (production batch number: #3033, Cell Signaling Technology, USA), NF-κB-p65 antibody (production lot number: #4746, Cell Signaling Technology, USA), IL-1β antibody (production batch number: ab9787, Abcam, UK), IL-18 antibody (production lot number: ab191860, Abcam, UK), caspase-1 antibody (production batch number: NBP1–45433, Novus Biological, USA), beta-actin mouse monoclonal antibody (production lot number: #4211, Cell Signaling Technology, USA), goat anti-rabbit secondary antibody (production batch number: AP132P, Merck Millipore, Germany), and goat anti-mouse secondary antibody (production lot number: AP124P, Merck Millipore, Germany).

### Construction of the PD mouse model

Mice in the Saline group were given 0.2 ml saline by gavage at 18:00 for 10 days and intraperitoneally injected with 0.2 ml saline on the 11th day. To mimic PD in vivo, mice in the PD group and MCC950 group were given estradiol (0.2 mg/ml, 0.2 ml/day) by gastric gavage at 18:00 for 11 days; this was used to synchronize the uterine cycles. The oxytocin receptor (OTR) overexpressed when activated by estrogen, and the concentrations of OTR were used to determine the sensitivity of endometrium to oxytocin stimulation [14, 15]. After gastric gavage of estradiol for 2 h, intraperitoneal injection of 0.2 ml MCC950 (2 mg/ml) was given to mice for 10 days in the MCC950 group. On the 11th day, both groups were given estradiol via intraperitoneal injection and 0.2 ml oxytocin (2 U/ml) within an hour [16]. Oxytocin is a small peptide hormone that stimulates uterine contractions by functionally coupling to the OTR to induce dysmenorrhea. This model of dysmenorrhea in mice has been in use for several years [17, 18]. All mice were fasted for 12 h before gavage. After daily intervention, they had free access to water and food.

### Measurement of writhing times

To assess the writhing response, we recorded 20-min writhing times and the writhing latency of mice. (Writhing response: the abdomen of the mouse was contracted and concave with straight hind limbs; writhing latency: from intraperitoneal injection of oxytocin to the first writhing reaction [19].)

### Thermal tail-flick test

The thermal tail-flick test was used to assess the nociceptive reflex response to thermal stimulus in treated animals. When the pain threshold of mice is exceeded, they use an effective tail flick escape [20]. The thermal tail-flick test of acute pain was performed on the 11th day after pharmaceutical injection. Each experimental mouse was placed in a fixator with the tail exposed. When it was stable, 1/3rd of the tail was placed in hot water at 52 °C. The tail-flick latency (from the tail entering the water to exiting) was measured with a stopwatch (accuracy: 0.01 s) for 4 consecutive times each with an interval of 1 min [21]. Each tail was measured in triplicate, and the average value was calculated.

### Enzyme-linked Immunosorbent assay (ELISA)

The levels of PGF_2α_ and PGE_2_ in uterine tissues were determined with an ELISA. The supernatant including total protein (TP) was obtained by homogenization and centrifugation from the same segment of ipsilateral uteruses of 5 mice randomly selected in each group. Each sample was incubated overnight with diluent and standard solution at 4 °C. Subsequently, the plate was washed and Ellman’s reagent was added, it was reacted at 4 °C in the dark for 60 min, and the absorbance value of each hole at 412 nm was measured. The concentration of PGF_2α_ was determined by the standard curve according to absorbance value of the standard hole and its corresponding concentration. The TP in collected supernatant was quantified by a BCA protein quantitative kit. Each sample was added with prepared standard compound based on the gradient method and incubated with working solution at 37 °C for 30 min, then the absorbance value of each hole at 412 nm was measured. The concentration of TP was determined by the standard curve according to absorbance value of the standard hole and its corresponding concentration. The ratio between PGF_2α_/PGE_2_ and TP indicated the content of PGF_2α_ in the supernatant of uterine homogenate. All procedures were according to the manufacturer’s protocol.

### Western blotting

The supernatant containing TP of the collected mouse uterine tissues was separated by homogenization using liquid nitrogen and 1 ml cold lysis buffer for 10 min, followed by centrifugation at 4 °C and 12,000 r/min for 10 min. To fully denature TP, the supernatant was added with sample buffer and heated in a boiling water bath for 10 min. The calculation and balance of protein concentration, together with the protein standard curve, was based on protein quantification via a BCA protein quantification kit. Samples were subjected to electrophoresis, membrane transfer, and blocking. Immunoblot analysis was performed with specific primary antibodies followed by secondary antibody: NLRP3 (1:1000), caspase-1 (1:500), IL-1β (1:1000), IL-18 (1:1000), phospho-NF-κB p65 (1:1000), NF-κB p65 (1:1000), COX-2 (1:1000), or β-actin (1:5000). Blots were incubated in enhanced chemiluminescence reagent, and exposed on radiographic film. The western blots were scanned and the density of the target bands was quantified by Quantity One software. The results for each protein were normalized against the intensity of β-actin in each sample. The relative protein content was expressed as arbitrary units.

### Statistical analysis

All data were analyzed using the SPSS version 22.0 statistical analysis package. All data are expressed as means ± standard deviation (S.D.) and were analyzed by two-tailed, unpaired, Student’s *t*-test or ANOVA if appropriate. A value of *p* < 0.05 was considered statistically significant.

## Results

### MCC950 inhibited the activation of NLRP3 inflammasomes in uterine tissues of mice with PD

**Fig. 1:**
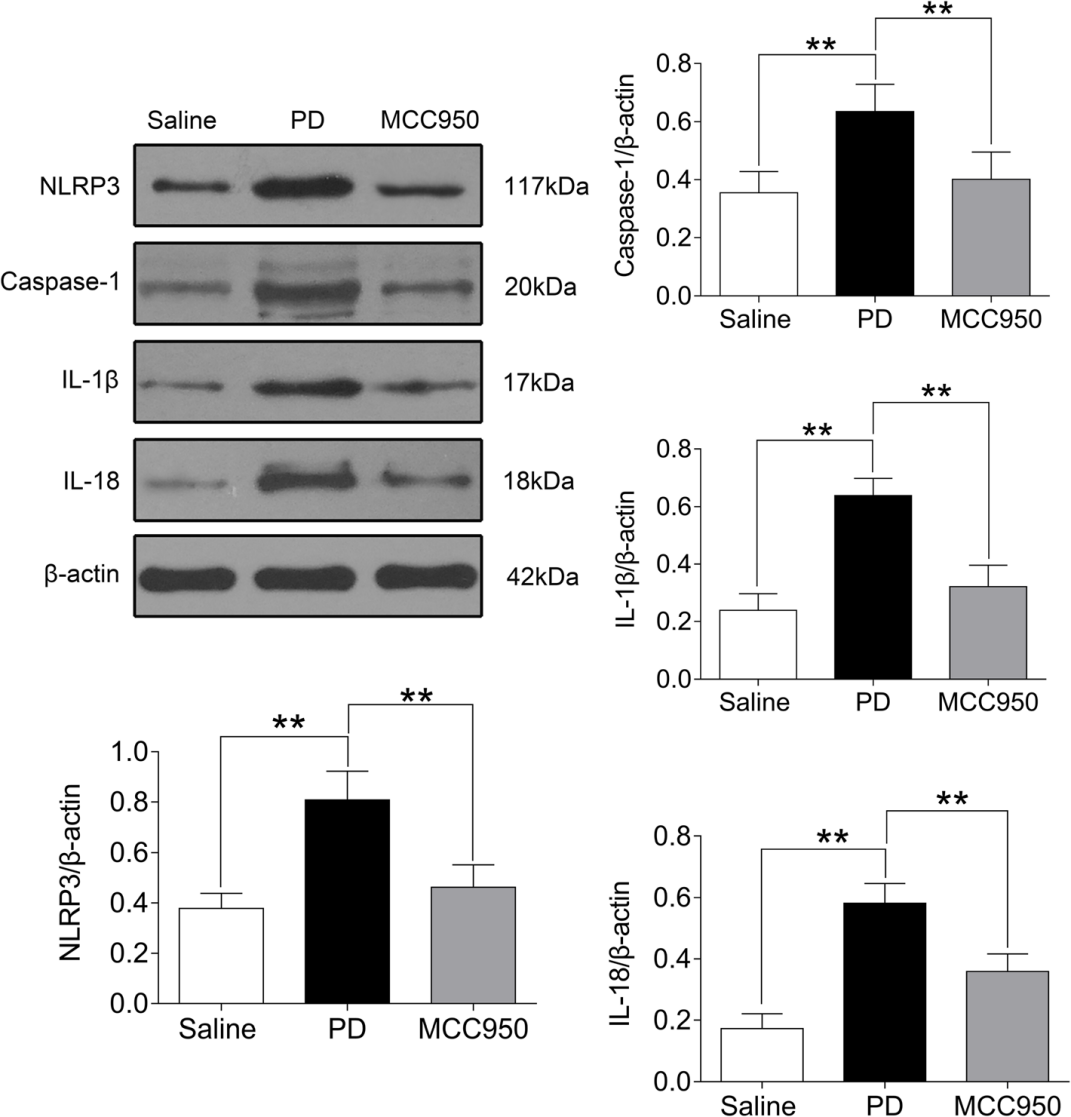
Comparison of NLRP3, cleaved-caspase-1 (p20) and activated forms of IL-1β and IL-18 proteins in mouse uteruses among groups. In the saline group, mice were treated with saline. In the PD group, estradiol (0.2 mg/ml) and oxytocin (2 U/ml) induced a significant decrease of NLRP3, caspase-1, IL-1β, and IL-18 proteins levels compared to the saline group. MCC950, an inhibitor of NLRP3, suppressed these protein levels according to western blotting. The data represent the mean ± SD (*n* = 5) of the relative protein content. The **asterisks** above the bars indicate significant differences compared to the PD group. **P < 0.01

Uterine tissues were examined by western blotting to detect the levels of NLRP3, cleaved-caspase-1 (p20) and activated forms of IL-1β, and IL-18. Western blotting analyses of the uterine tissues of saline-injected mice showed low levels of NLRP3, cleaved-caspase-1 (p20) and activated forms of IL-1β, and IL-18. In contrast, within 20 min after oxytocin injection, the levels of NLRP3, cleaved-caspase-1 (p20), and activated forms of IL-1β and IL-18 in uterine tissues of the PD group increased significantly (Fig. 1, *P* < 0.01), but they did not increase in the MCC950 group (Fig. 1, *P* < 0.01).

### MCC950 reduced writhing times and prolonged writhing latency and tail-flick latency in mice with PD

**Fig. 2:**
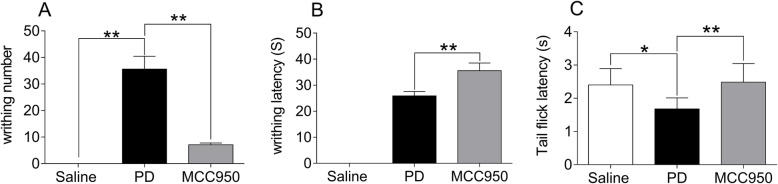
Comparison of writhing number in 20 min, writhing latency, and tail-flick latency of mice among groups. The mice were pretreated with saline, or estradiol (0.2 mg/ml) and oxytocin (2 U/ml) to build a PD model, or MCC950 (2 mg/ml, an NLRP3 inhibitor) in the presence of estradiol and oxytocin. All tests were double-blinded. **a** the typical writhing response corresponds to the writhing number. **b** time from intraperitoneal injection of oxytocin to the first writhing reaction. **c** One thirdx of the tail was placed in hot water at 52 °C, and the time was measured from the tail entering the water to exiting. Data are expressed as means ± SD (*n* = 10). *P < 0.05, **P < 0.01 compared to the PD group

To investigate whether NLRP3 inflammasome activation promotes PD pain, we first examined the effect of an NLRP3 inhibitor on PD induced by estradiol and oxytocin in mice. There was no writhing response in the Saline group, while mice in the PD group and MCC950 group showed alert writhing responses. Within 20 min after oxytocin injection, the writhing response numbers of the PD group significantly increased (Fig. 2a, *P* < 0.01) with abbreviated tail-flick latency (Fig. 2*c, P* < 0.01) compared with the Saline group. Compared with the PD control group, the writhing numbers of the MCC950 group were significantly decreased (Fig. 2a*, P* < 0.01), and the writhing latency and tail-flick latency were increased (Fig. 2b and Fig. 2*c, P* < 0.01) within 20 min (Fig. 2).

### MCC950 inhibited production of PG in uterine tissues of mice with PD

**Fig. 3:**
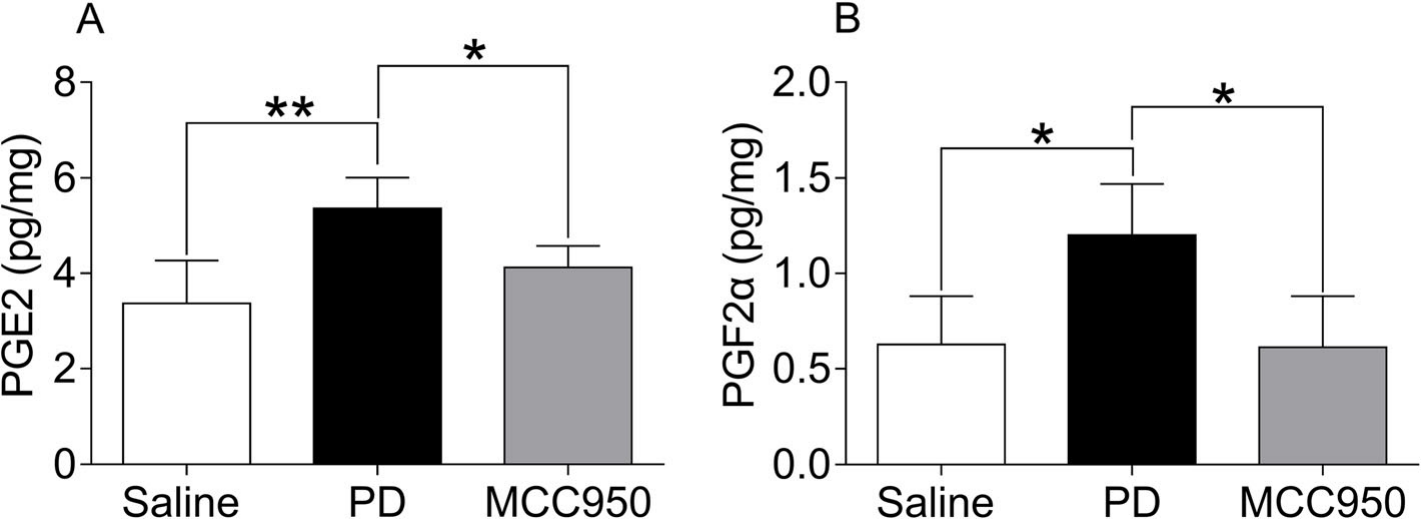
Comparison of PGE_2_ and PGF_2α_ in mouse uteruses among each group. The mice were pretreated with saline, or estradiol (0.2 mg/ml) and oxytocin (2 U/ml) to build a PD model, or MCC950 (2 mg/ml, an NLRP3 inhibitor) in the presence of estradiol and oxytocin. **a** Levels of PGE_2_ and **b** levels of PGF_2α_ were assayed by **ELISA**. The results are presented as means ± SD (*n* = 5). *P < 0.05, **P < 0.01 compared to the PD group

PG is known to play a role in PD as a mediator of inflammation. We next tested whether an NLRP3 inhibitor can regulate PG in the presence of PD. Compared with the Saline group, PGF_2α_ and PGE_2_ in the PD group were significantly increased (Fig. 3, *P* < 0.05 or *P* < 0.01). Compared with the PD group, the levels of PGF_2α_ and PGE_2_ in the MCC950 group were decreased (Fig. 3, *P* < 0.05).

### MCC950 decreased the level of COX-2 in uterine tissues of mice with PD

**Fig. 4:**
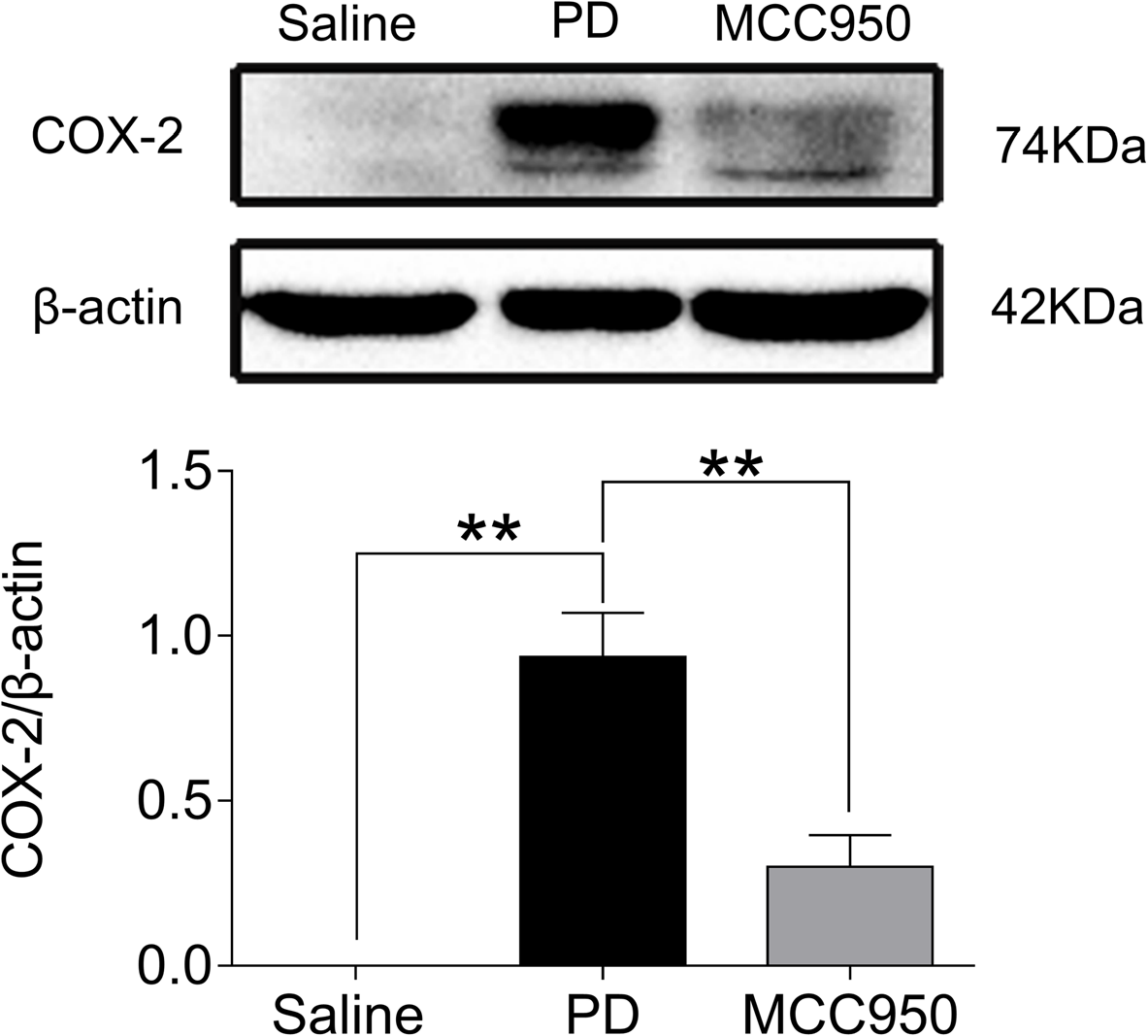
Comparison of COX-2 protein expression in mouse uteruses among groups. No upregulation of COX-2 expression occurred in the saline group (β-actin used as control). Western blot analyses of COX-2 expression in mice co-stimulated with estradiol (0.2 mg/ml) and oxytocin (2 U/ml) in the PD group. A significant decrease of COX-2 protein expression occurred compared to the saline group. MCC950, an inhibitor of NLRP3, suppressed COX-2 protein expression. Data are expressed as means ± SD (*n* = 5). **P < 0.01 compared to the PD group

Next, we determined the effect of NLRP3 inflammasome inhibition on COX-2 as an upstream regulatory protein of PG in PD mice. As expected, the protein level of COX-2 in the uterine tissues of mice in the Saline group was very low and could not be detected by western blotting. Compared with the Saline group, the COX-2 protein level in the uterine tissues of the PD group was significantly increased (Fig. 4, *P* < 0.01). Subsequently, we confirmed that the levels of COX-2 were decreased significantly in the MCC950 group compared with those of PD group (Fig. 4, *P* < 0.01).

### MCC950 suppressed activation of the NF-κB pathway in uterine tissues of mice with PD

**Fig. 5:**
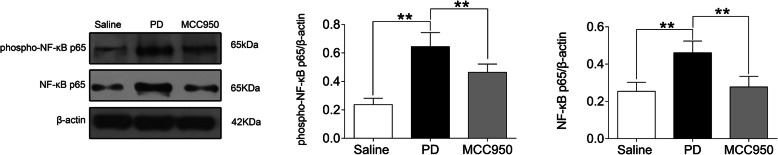
Comparison of phospho-NF-κB p65 and NF-κB p65 protein expression in mouse uteruses among groups. In the PD group, estradiol (0.2 mg/ml) and oxytocin (2 U/ml) induced a significant decrease of phospho-NF-κB p65 and NF-κB p65 protein expression compared to the saline group. MCC950, an inhibitor of NLRP3, suppressed these increases in protein expression according to western blotting The data represent the means ± SD (*n* = 5) of the relative protein content. **P < 0.01 compared to the PD group

To confirm whether the effect of NLRP3 inflammasomes on PD-induced pain was related to NF-κB p65, we investigated NF-κB p65 phosphorylation by western blotting. Compared with the Saline group, the protein levels of phospho-NF-κB p65 and NF-κB p65 in the PD group were significantly increased (Fig. 5, *P* < 0.01). Compared with the PD group, the protein levels of phospho-NF-κB p65 and NF-κB p65 in the MCC950 group were decreased significantly (Fig. 5, *P* < 0.01).

## Discussion

It has become clear that the NLRP3 inflammasome is involved in a broad spectrum of gynecological diseases such as cervical cancer, preterm labor, postpartum inflammation, and mycoplasma and chlamydia infection [22, 23]. Our study found that NLRP3, cleaved-caspase-1 (p20) and activated forms of IL-1β and IL-18 were dramatically increased in mice with PD, which could be reversed by intervention with MCC950, a specific inhibitor of NLRP3 that only inhibits the activation of caspase-1 and the processing of IL-1β and IL-18 [24]. Recent reports show that MCC950 dose-dependently decreases the efficiency of NLRP3 NACHT domain immunoprecipitation, which agrees with our results [25, 26]. These results suggested that the NLRP3 inflammasome is expressed and activated in the uteruses of PD mice, which may offer substantial promise in developing new therapeutics for PD. Emerging evidence suggests that NLRP3 inflammasome activation occurs in endometritis cells, while inhibiting NLRP3 inflammasomes generates a significant ameliorative role in the pathological process of endometritis [27]. Moreover, the activation of the NLRP3 inflammasome aggravates the degree of inflammatory infiltration in gynecological diseases such as endometritis and pelvic inflammatory disease, which indicates that therapeutic interventions via the NLRP3 inflammasome may be a novel strategy for treating PD [28, 29]. Therefore further investigation is warranted to explore the role of the NLRP3 inflammasome in PD.

In our study, mice in the PD group presented obvious writhing responses via administration of oxytocin. In contrast, writhing times were significantly decreased and tail-flick latency was noticeably elongated in the MCC950 group. The writhing response measures pain in a direct way, and the tail-flick latency indirectly evaluates pain threshold changes [30, 31]. The results suggested that NLRP3 suppression exerted an important role in both the anti-inflammatory and analgesic response against PD. Further, we concentrated on the NF-κB/COX-2/PG pathway to elucidate the potential mechanism of PD.

The PGF_2α_ and PGE_2_ levels of mice uteruses in the PD group robustly increased, while they significantly decreased through MCC950 intervention. It has been reported that both PGF_2α_ and PGE_2_ are the primary members of prostaglandins that play a critical role in the inflammatory process. The levels of PGF_2α_ and PGE_2_ in the uterine tissues and peripheral blood of PD patients are higher than normal [2]. Additionally, numerous studies have illustrated that high levels of PGF_2α_ lead to pathological pain responsible for uterine contraction, hematological alterations, and the accumulation of acidic metabolites [32]. In this study, we found that PGF_2α_ and PGE_2_ in uterine tissues of PD mice markedly decreased by inhibiting NLRP3 inflammasomes.

COX-2, an important limiting rate enzyme participating in PG synthesis, exhibits a tight relationship with uterine inflammation [33], while upstream, NF-κB mediates the expression and activation of COX-2. We measured the levels of NF-κB and COX-2 in the uterine tissues of mice after MCC950 intervention. The results showed that COX-2 in the Saline group was scarce, while it significantly increased in the uterine tissues of PD mice. Furthermore, MCC950 intervention reversed the potent increase of COX-2 protein levels in PD mice. Under physiological conditions, COX-2 exists at a very low level, but it is elevated by inflammatory stimuli. Studies have shown that the expression of COX-2 goes up before menstruation, which catalyzes arachidonic acid into active PG, resulting in spasmodic pain. Nonsteroidal anti-inflammatory drugs, such as ibuprofen, play a profound role in treating the symptoms of PD via COX-2 inhibition with a subsequent decrease of PG [34].

As an important transcriptional regulator, NF-κB plays a crucial role in PD via regulation of COX-2 [35]. In general terms, the phosphorylation of the NF-κB subunits has a profound effect on the function of NF-κB and promotes its translocation [36, 37]. As detected by western blot analysis, the levels of phospho-NF-κB p65 and NF-κB p65 significantly increased in the uterine tissues of mice with PD. It is well known that NF-κB is needed during the priming phase to induce NLRP3 and pro-IL1B expression [38, 39]. However, a report on nonalcoholic fatty liver disease showed that MCC950 can reduce inflammation and NF-κB activation by inhibiting NLRP3 [40]. It has been also shown that IL-1β, a downstream inflammatory factor of NLRP3, can promote the expression of NF-κB by phosphorylating serines 316, 529 and 536 of the p65 subunit to regulate the target gene [41]. This is consistent with our results. MCC950 intervention strongly attenuated the activation of phospho-NF-κB p65 and NF-κB p65 in PD mice, which indicated that the NF-κB pathway plays a pathogenic role in PD, and that inhibiting NLRP3 inflammasomes could downregulate NF-κB activation. Previous studies have shown that some NF-κB site sequences are located in the flanking region of the 5′ end of the COX-2 coding sequence. NF-κB induce the activation of COX-2 by binding to these sites, which indicates that NF-κB is a key factor that regulates COX-2 [42, 43]. It has also been reported that NF-κB induces the activation of COX-2 genes in primary dysmenorrhea [44], and some studies have shown that the NLRP3 inflammasome upregulates NF-κB activation and promotes COX-2 expression [45]. Moreover, it has been reported that NLRP3 inflammasomes could be responsible for mediating the albumin effect on activating the COX-2/mPGES-1/PGE2 cascade, but the role of NF-kB in this case is not known [12]. The effect of NLRP3 on COX-2 is not clear, but we think that NLRP3 regulates COX-2 through the NF-κB pathway, and the NLRP3 inflammasome might be involved in the pathological progression of PD through the NF-κB/COX-2/PG pathway. In addition, it has been reported that COX-2 has an effect on NLRP3 and NLRP3 inflammasome activation that is regulated by dephosphorylation of COX-2 modified by PP2A [46], and COX-2 mediateds the enhancement of lipopolysaccharide-induced pro-IL-1β and NLRP3 expression by increasing NF-kB activation and enhancing caspase-1 activation by increasing damaged mitochondria [47]; these effects are inhibited by the COX-2 inhibitor celecoxib. It has also been s reported that PTUPB, a dual COX-2 and sEH inhibitor, also inhibits the activation of NLRP3 inflammasomes [48, 49]. We speculate that MCC950 might reduce inflammation by blocking the positive feedback of COX-2 on NLRP3 inflammasomes. However, NLRP3 inflammasome activation is inhibited through its COX-2 metabolite, PGE2-EA, and this effect is inhibited by celecoxib [50]. At present, the relationship between NLRP3 inflammasomes and COX-2 is not clear in PD and needs further research in the future. This study is potentially limited in that we mainly measured proteins levels by western blotting and ELISA rather than gene and transcription levels. However, since protein expression is the outcome of gene. Activity, we can preliminarily determine the role of these proteins and corresponding genes in PD by measuring protein levels and deducing its mechanism.

## Conclusions

In summary, activation of NLRP3 inflammasomes induced deleterious effects in PD mice, and therapeutic interventions targeting NLRP3 inflammasomes via the NF-κB/COX-2/PG pathway mitigated the agonizing pain responsible for PD. This novel finding of an inflammatory effect of NLRP3 inflammasomes in PD contributes to our understanding of the disease and provides a promising new target for PD intervention.

## Data Availability

The data that support the findings of this study are available from the corresponding author upon request.

## Abbreviations

PD: Primary dysmenorrhea;
NLRP3: Nucleotide-binding oligomerization domain-like receptor protein 3;
Caspase-1: Cysteinyl aspartate-specific protease 1;
IL-1β: Interleukin-1β;
IL-18: Interleukin-18;
NF-κB: Nuclear factor kappa B;
COX-2: Cyclooxygenase-2;
PGE_2_: Prostaglandin E_2_;
PGF_2α_: Prostaglandin F_2_ alpha;
ELISA: Enzyme-Linked Immunosorbent Assay;
TP: Total protein;
